# Targeted autonomic testing for radiation‑induced baroreflex failure in head and neck cancer survivors: index case and early program experience

**DOI:** 10.1186/s40959-026-00501-2

**Published:** 2026-05-29

**Authors:** Efthymios Triantafyllou, Ingrid González Flores, Cezar Iliescu, Anita Deswal, Elie Mouhayar, Clifton D. Fuller, Efstratios Koutroumpakis

**Affiliations:** 1 Department of Cardiology, Division of Internal Medicine, The University of Texas MD Anderson Cancer Center, 1515 Holcombe Blvd # 1451, Houston, TX 77030 USA; 2 Department of Radiation Oncology, The University of Texas MD Anderson Cancer Center, Houston, TX US

**Keywords:** Autonomic testing, Baroreflex failure, Autonomic dysfunction, Head and neck cancer, Radiation therapy

## Abstract

Radiation‑induced afferent baroreflex failure (R‑ABF) is a highly morbid late effect of neck irradiation in head and neck cancer survivors, and is frequently underrecognized due to nonspecific, fluctuating symptoms. We report an index case in which systematic autonomic evaluation—including adrenergic and cardiovagal assessment—enabled objective diagnosis, severity grading, while physiology‑directed therapy resulted in meaningful symptomatic improvement. The diagnostic trajectory highlights recurrent challenges: prolonged latency from symptom onset to recognition, therapeutic complexity in the setting of cardiovascular comorbidities, and limited access to standardized autonomic testing. In response, we have developed the BARO‑CARE Program, a structured pathway integrating comprehensive autonomic profiling to streamline diagnosis, guide individualized management, and support longitudinal monitoring. This case and early program experience emphasize that even chronic R‑ABF remains therapeutically modifiable when approached with pathophysiologically‑guided care. Programmatic, multidisciplinary models may reduce diagnostic delays and improve outcomes as the survivor population grows.

**Supplementary Information:**

The online version contains supplementary material available at 10.1186/s40959-026-00501-2.

## Introduction

Radiation-induced afferent baroreflex failure (R-ABF) is a debilitating late complication of radiation therapy (RT) for head and neck cancer (HNC) [1]. Although increasingly recognized, it remains substantially underdiagnosed, with many patients experiencing delays of several years before a definitive diagnosis is made [2]. Advances in oncologic treatments, along with the rising incidence of human papillomavirus–associated oropharyngeal cancer, have shifted the HNC population toward younger individuals with fewer comorbidities, who now survive for decades after RT [3]. As survival improves, long‑term treatment‑related sequelae, such as autonomic dysfunction, have become increasingly clinically relevant and increasingly impactful on quality of life.

R-ABF typically manifests years after cervical therapeutic irradiation and is characterized by extreme blood pressure lability, paroxysmal hypertensive crises, orthostatic hypotension, disabling lightheadedness, syncope, and arrhythmias [4]. These symptoms can significantly impair daily function and quality of life and can even be associated with serious bodily injury following syncope or falls. Despite its clinical impact, diagnosis and management remain challenging, in part because the condition spans multiple specialties and presents with heterogeneous autonomic features. Although autonomic testing has emerged as a valuable tool for characterizing baroreflex dysfunction, substantial gaps remain in its availability and in systematic evaluation, severity grading, risk stratification, and evidence‑based treatment pathways [5]. These limitations underscore the need for prospective studies, prediction tools, and standardized anticipatory management algorithms for this growing population of HNC survivors. In this report, we present an index case of R-ABF illustrating noninvasive evaluation of cardiovascular autonomic dysfunction, severity grading, and successful medical management. We further describe our early experience from the Baroreflex Assessment & Rehabilitation for Oncology‑Related Effects (BARO‑CARE) Program, which integrates targeted noninvasive autonomic testing for patients with suspected R‑ABF and implements treatments aimed at the underlying pathophysiology and baroreceptor impairment. Across our initial cohort, we highlight shared clinical features, recurring diagnostic patterns, and emerging insights from this evolving cardio‑oncology survivorship initiative.

## Index case presentation

A 54‑year‑old man initially presented with stage T1 Nx cM0 squamous cell carcinoma (SCC) of the tongue. He underwent surgery with left partial glossectomy with clean surgical margins and no residual disease. Twenty years later, at age 74, he developed a locally recurrent, moderately differentiated SCC with involvement of cervical lymph nodes, staged as T3 N2a M0. He subsequently underwent a level I–III neck dissection followed by postoperative volumetric modulated arc therapy (VMAT) of 60 Gy in 30 fractions, achieving complete remission. Four years after completion of RT, he began experiencing recurrent episodes of lightheadedness. Clinical evaluation revealed profound labile blood pressure and orthostatic hypotension.

Given his history of hypertension, his fluctuating blood pressure episodes were initially managed through adjustments of his antihypertensive regimen, with minimal improvement. One year later, his course was complicated by a non–ST‑elevation myocardial infarction, for which he underwent coronary revascularization. Subsequently, he developed a high burden of ventricular ectopy and atrial flutter/fibrillation, requiring radiofrequency ablation. This was followed by progressive bradycardia necessitating permanent pacemaker implantation. Despite appropriate management of his cardiovascular comorbidities and multiple modifications of his medications, he continued to experience marked and symptomatic blood pressure instability, with systolic values ranging from the 70s to over 215 mmHg. After nearly two years without a unifying diagnosis, he presented to the emergency department with altered mental status and expressive aphasia during a hypertensive crisis (193/95 mmHg). Brain imaging was unremarkable, and his symptoms improved with intravenous hydralazine. Following this event and upon discharge, he was referred to our BARO‑CARE program for evaluation.

Upon evaluation in our BARO‑CARE Clinic, the patient reported daily episodes of lightheadedness and presyncope associated with systolic blood pressure readings below 100 mmHg. At the time of presentation, he was taking propranolol 10 mg twice daily. His clinic blood pressure was markedly elevated at 185/84 mmHg, with a heart rate of 79 bpm. Laboratory testing showed normal electrolytes and renal function. Electrocardiography demonstrated an atrial‑paced rhythm at 65 bpm, and transthoracic echocardiography revealed normal biventricular function with no structural abnormalities. A carotid duplex ultrasound demonstrated non‑hemodynamically significant atherosclerotic plaque with less than 50% stenosis of the left internal carotid artery. Given his history of cervical irradiation and the constellation of fluctuating blood pressure measurements and autonomic symptoms, R-ABF was strongly suspected, and he was referred for cardiovascular autonomic testing. Formal orthostatic blood pressure measurements were deferred to the subsequent standardized autonomic evaluation to ensure reproducible, beat-to-beat hemodynamic assessment.

**Fig. 1:**
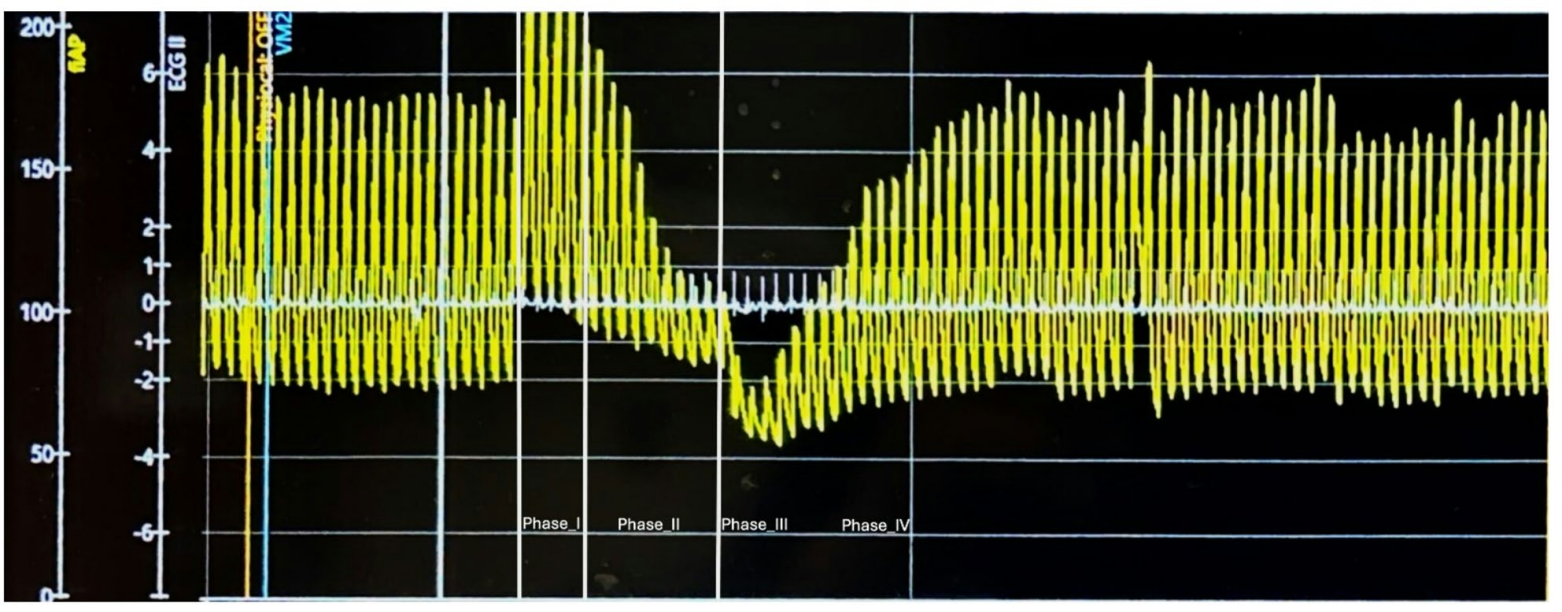
Valsalva Maneuver. In this figure, the four distinct phases of the Valsalva maneuver can be identified. Notably, phase II late and phase IV are absent and blood pressure recovery time is 14 s, indicating moderate adrenergic dysfunction

**Fig. 2:**
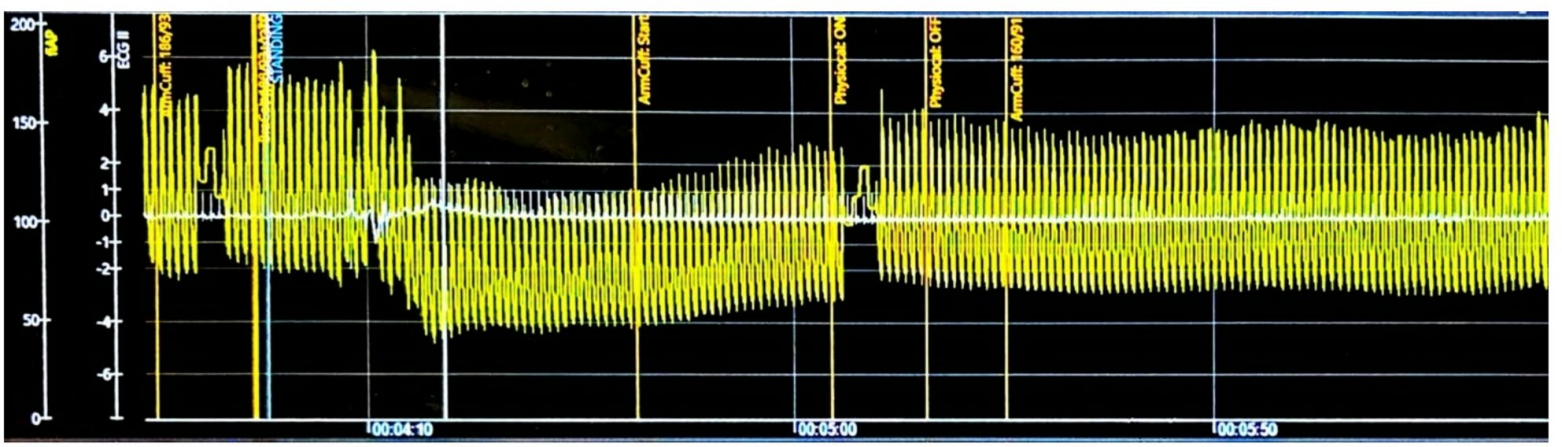
Active standing maneuver. Immediate drop in systolic and diastolic blood pressures greater than 20 mmHg and 10 mmHg respectively were noted upon standing. The blood pressure remained persistently low throughout the standing period without complete recovery. These findings are consistent with orthostatic hypotension

Cardiovascular autonomic testing was performed using the validated, noninvasive hemodynamic Finapres NOVA Plus system [6]. Findings were graded according to the modified Composite Autonomic Severity Score (CASS), which evaluates adrenergic and cardiovagal function on 0–4 and 0–3 scales, respectively, with 0 indicating normal function [7]. The BP at baseline was 179/92 mmHg, with a mean BP of 121 mmHg and a pulse pressure of 87 mmHg. During phase II early of the Valsalva maneuver, BP dropped to 126/106 mmHg, with a mean BP of 112 mmHg and a marked narrowing of pulse pressure from 87 to 20 mmHg, accompanied by absent phase II late and a pressure recovery time of 14 s - meeting the criteria for a CASS adrenergic score of 2 out of 4, indicating moderate adrenergic dysfunction (Fig. 1). Cardiovagal assessment could not be performed due to persistent atrial‑paced rhythm during the study, limiting the ability to calculate the Valsalva ratio or heart rate variability during the deep breathing maneuver. Additional abnormalities included supine hypertension (BP 179/92) and marked orthostatic hypotension (Supine BP 183/92 mmHg, standing BP at 1-minute 136/75 mmHg, at 3-minutes 157/78 mmHg) (Fig. 2). During the cold pressor maneuver, an exaggerated hypertensive response was observed, with systolic blood pressure rising by 27 mmHg. Together, these findings supported the diagnosis of afferent baroreflex failure and allowed for objective severity grading.

For management, the patient was monitored for one week to establish baseline symptom patterns and blood pressure variability. He was then initiated on midodrine (alpha-1 adrenergic agonist) and guanfacine (centrally acting alpha-2 adrenergic agonist), with close follow‑up, home blood pressure monitoring, and gradual dose adjustments over the subsequent three months. However, he developed worsening obstructive urinary symptoms attributed to midodrine, leading to its discontinuation. Atomoxetine (serotonin-norepinephrine reuptake inhibitor) was then tried as an alternative to midodrine, but it did not result in symptomatic improvement and was subsequently discontinued. Following treatment optimization for his obstructive uropathy, the patient was re‑challenged with low‑dose midodrine; however, he developed a rash consistent with a suspected allergic reaction, prompting permanent discontinuation. Given the severity of his symptomatic hypotension episodes, he was started on low‑dose fludrocortisone at 0.1 mg daily, with careful consideration of potential side effects in the context of his cardiovascular history. To better target the timing of his autonomic instability, guanfacine administration was shifted to the early afternoon. Ultimately, a stable therapeutic regimen was established, leading to infrequent hypotensive episodes and marked symptomatic improvement. His current pharmacologic therapy consists of fludrocortisone 0.1 mg once daily on awakening and guanfacine 1.5 mg administered in the early afternoon.

At his most recent follow‑up, ten weeks after the final adjustment of his regimen, the patient reported near‑complete resolution of symptoms, with systolic blood pressure predominantly in the 100–150 mmHg range (compared with 70–210 mmHg prior to treatment). His former daily pattern of near‑syncope followed by episodes of markedly elevated blood pressure had decreased to only three mild, asymptomatic incidents over the entire 10‑week period. Hypertensive crises, which previously occurred almost daily, were reduced to approximately once weekly with the new treatment regimen. Hypotensive episodes with near-syncope, previously occurring daily, decreased to three mild, asymptomatic events over the entire 10-week follow-up period. He also reported substantial improvement in quality of life, noting that his daily activities were no longer limited by blood pressure instability.

## The baroreflex assessment & rehabilitation for oncology‑related effects (BARO‑CARE) program

As a tertiary cancer center with a large population of patients and survivors with HNC, we have observed an increasing number of long‑term survivors presenting with unexplained labile blood pressure, resting tachycardia, recurrent syncope, and orthostatic symptoms. This emerging clinical pattern prompted the development of a structured initiative—the BARO‑CARE Program—to establish timely diagnosis, objectively grade severity, and guide management based on underlying pathophysiology rather than empirical treatment alone.

We acquired FDA‑approved, validated equipment for noninvasive cardiovascular autonomic testing (Finapres NOVA Plus), and our team underwent dedicated training to perform the maneuvers and interpret the results. Guided by published evidence and the American Autonomic Society expert consensus on autonomic evaluation, we developed an institutional protocol for standardized noninvasive autonomic testing [7]. Prior to testing, patients referred to the BARO‑CARE Program undergo screening. Detailed medical history and current medications are recorded. Patients are instructed to avoid caffeine and alcohol for at least 12 h and to refrain from solid food for 8 h while maintaining hydration with non‑caffeinated, non‑alcoholic beverages. Upon arrival, an intravenous line is placed, and after a minimum of 10 min of supine rest, a baseline blood sample is drawn for catecholamine measurement.

All maneuvers are performed in the supine position. Testing begins with a standardized Valsalva maneuver, during which patients generate ≥ 40 mmHg of expiratory pressure for 15 s. Blood pressure and heart rate responses are recorded across the four phases of the maneuver, and findings are graded using the validated CASS scoring system, providing a comprehensive assessment of both adrenergic and cardiovagal function. Next, a deep breathing maneuver is performed to assess heart rate response to deep breathing (HRDB), a key contributor to the cardiovagal component of the Composite Autonomic Severity Score (CASS). HRDB is selected because it is less affected by baseline heart rate and beat‑to‑beat variability compared with the expiratory‑to‑inspiratory ratio (E: I) or mean circular resultant (MCR). The Valsalva and deep breathing maneuvers are performed three times with two‑minute rest intervals. This is followed by active standing, in which the patient stands upright and remains still for five minutes. Active standing was chosen over tilt‑table testing due to its reproducibility and practicality, despite eliciting a somewhat smaller orthostatic stimulus. The 30:15 ratio, which assesses the cardiovagal function through heart rate response to standing, is not calculated, as cardiovagal assessment is more accurately characterized by HRDB and the Valsalva maneuver. At the end of the standing period, a second blood sample is obtained for repeat catecholamine analysis. Supine and standing norepinephrine levels provide an additional quantitative measure of sympathetic activity and orthostatic response. Finally, a cold pressor test is performed to help differentiate afferent from efferent baroreflex dysfunction. The patient immerses the forearm, palm facing upward, in 4 °C ice water for 60–90 s, during which blood pressure responses are recorded.

**Fig. 3:**
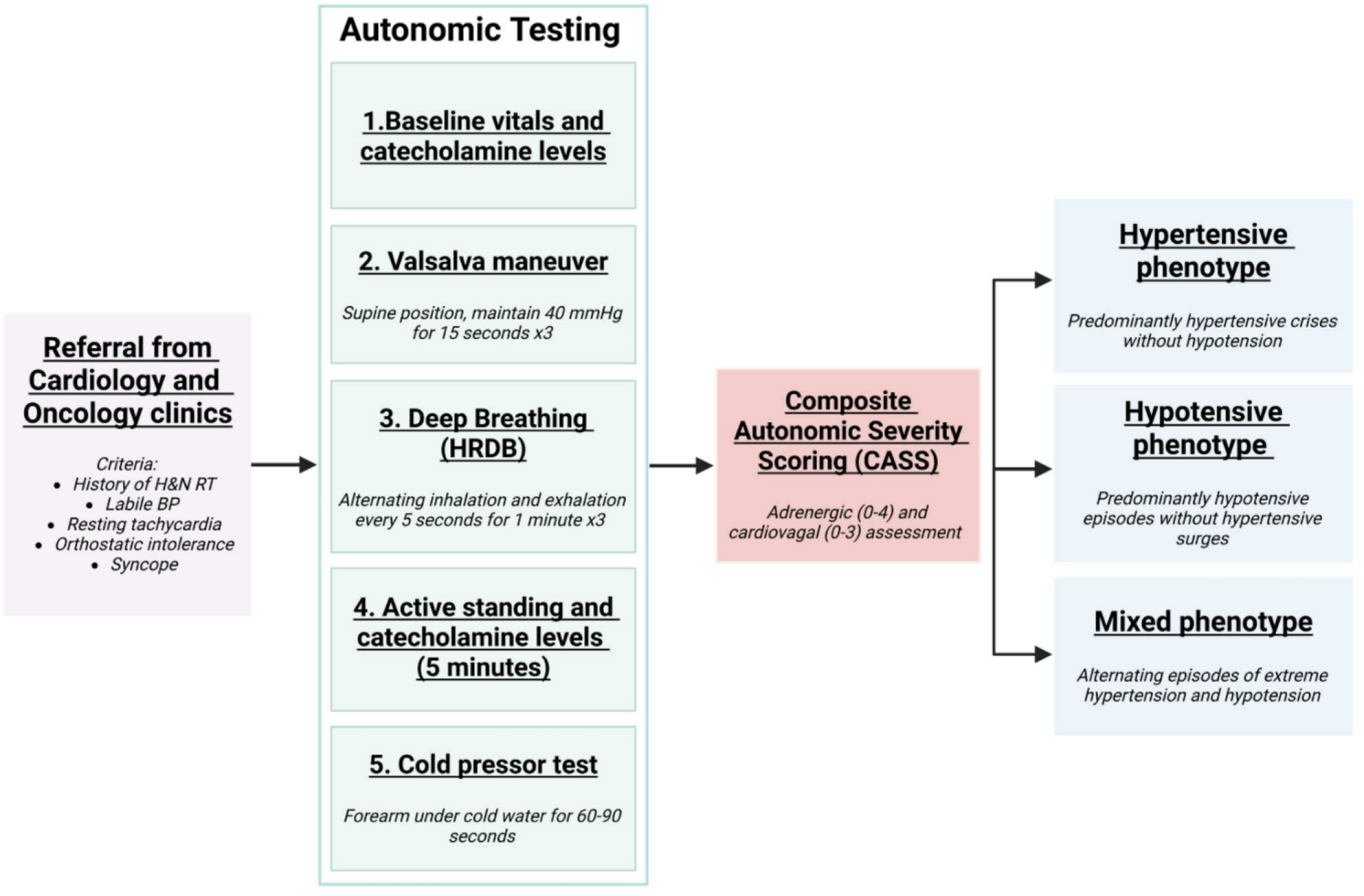
The BARO-CARE Program: autonomic testing protocol and diagnostic pipeline. Patients presenting to cardiology or oncology clinics with symptoms consistent with R-ABF are referred for structured autonomic evaluation. Testing comprises four sequential maneuvers - Valsalva, deep breathing, active standing, and cold pressor test - with plasma catecholamine levels measured at baseline and five minutes after standing. Results are integrated into the Composite Autonomic Severity Score (CASS) and guide classification into one of three clinical phenotypes based on the predominant hemodynamic pattern

Based on the autonomic testing results and CASS grading, patients are classified into one of three clinical phenotypes: a hypertensive phenotype characterized by predominant hypertensive crises, a hypotensive phenotype characterized by recurrent hypotensive episodes, and a mixed phenotype with alternating extremes of hypertension and hypotension. The BARO-CARE Program clinical pathway is summarized in Fig. 3. The complete autonomic testing protocol is provided in the Supplementary Material.

To date, we have evaluated 10 HNC survivors treated with neck irradiation. The predominant indications for testing were frequent lightheadedness and labile blood pressure. Nine of the ten patients demonstrated evidence of autonomic dysfunction. The median time from completion of RT to R‑ABF diagnosis was 10 years. In this cohort, autonomic impairment involved both adrenergic and cardiovagal domains, with a trend toward more pronounced adrenergic dysfunction. Three patients had also received neurotoxic chemotherapy as part of their cancer treatment (Table 1).

**Table 1:** Patient characteristics

Patient	Age at Radiation	Age at symptom manifestation	Age at testing & diagnosis		Neck dissection (+) / Neurotoxic chemotherapy (+)	Radiation exposure to carotid (EQD2) (Gy)	Composite autonomic severity score (Adjusted)	Afferent (+) / Efferent (-)
1	69	74	76		-/-	70	Moderate (3)	-
2	53	75	76		+/-	110.64	Moderate (4)	+
3	53	56	69		+/+	71.63	Moderate (3)	+
4	61	74	75		+/+	84.75	Mild/ Moderate (2)	+
5	74	78	80		-/-	60	Moderate/ Severe (5)	+
6	72	80	83		+/-	71.68	Moderate (3)	+
7	66	78	78		-/+	60	Mild (1)	+
8	89	89	89		-/-	60	Mild (2)	+
9	56	57	57		+/-	68.64	None (0)	+
10	67	73	76		-/-	50	Mild (1)	+

Moving forward, the BARO‑CARE program will continue to expand screening efforts for individuals with suspected R‑ABF, characterize the severity and phenotype of autonomic dysfunction, and guide tailored medical therapy informed by autonomic testing. Our long‑term goal is to identify early signs of autonomic injury when physiologic recovery may still be possible and to evaluate preventive strategies that mitigate baroreceptor damage in HNC survivors.

## Discussion

This report highlights an index case of R‑ABF in which systematic autonomic testing enabled objective diagnosis and severity grading, and targeted therapy led to meaningful symptom relief. The clinical course underscores three recurring challenges: (i) prolonged diagnostic latency due to nonspecific, fluctuating presentations; (ii) therapeutic complexity in the setting of coexisting cardiovascular disease; and (iii) the central role of standardized autonomic evaluation in guiding treatment selection and timing. In parallel, we established the BARO‑CARE Program to provide a structured clinical pathway for HNC survivors with prior neck irradiation.

R-ABF results from fibrosis of the carotid sinus and damage to the afferent fibers of the glossopharyngeal and vagus nerves, impairing the ability of baroreceptors to sense and transmit changes in arterial pressure to the central nervous system [1, 8]. The resulting hemodynamic instability - hypertensive surges, tachycardia, orthostatic hypotension and extreme blood pressure lability - is multifactorial, likely reflecting contributions from unregulated sympathetic activation, hypovolemia, medication effects and radiation-induced vascular damage. In cancer patients, prolonged bed rest, such as during postoperative recovery or extended hospitalizations for oncologic treatment, may further compound baroreflex dysfunction through deconditioning, reduced plasma volume, cardiac atrophy, and increased sympathetic activation mediated by GABAergic inhibition of the rostral ventrolateral medulla [9]. A further potential mechanism involves the disruption of rhythmic cardiovascular oscillations around 0.1 Hz, the low-frequency band that reflects baroreflex-mediated sympathetic modulation of vascular tone, through which baroreceptors coordinate synchronized fluctuations of blood pressure, heart rate, and muscle sympathetic nerve activity (MSNA) during orthostatic stress [10]. Furlan et al. first described the complete absence of this rhythmicity in baroreflex failure, with sympathetic discharge becoming disorganized and uncoupled from arterial pressure despite high tonic MSNA and elevated catecholamines [10]. This has since been validated in healthy volunteers progressing to presyncope during the European Space Agency Medium-Term Bed Rest Study, demonstrating that the pattern of sympathetic discharge, and not merely its quantity, determines the effectiveness of vasoconstriction during orthostatic challenge [11, 12].

Despite its significant clinical impact, R-ABF remains frequently underrecognized, as its presentations are easily attributed to more common conditions encountered across primary care, cardiology, and neurology [1, 13]. Awareness of R‑ABF among frontline clinicians remains limited, and access to comprehensive autonomic testing is scarce. This leads to fragmented evaluations and empiric medication adjustments that may inadvertently worsen hemodynamic lability. Consistent with prior reports, both our index case and early program experience demonstrate prolonged intervals from symptom onset to diagnosis [5]. These observations highlight the need for clear referral triggers (e.g., unexplained blood pressure lability or syncope in HNC survivors with prior neck irradiation) and ready access to autonomic laboratories capable of performing Valsalva‑based assessment and complementary maneuvers.

Against this backdrop, programmatic approaches such as BARO‑CARE can reduce diagnostic uncertainty and facilitate earlier detection of R‑ABF. A standardized protocol - including Valsalva and deep‑breathing maneuvers, active standing, cold pressor testing, and catecholamine sampling - supports consistent diagnosis and severity grading (e.g., using a modified CASS), enables longitudinal tracking, and anchors therapeutic decisions in physiology rather than empiricism [4, 14]. The rising prevalence of HPV-associated oropharyngeal cancer, now the most common type of HNC, has shifted the affected population toward younger individuals who lack traditional risk factors such as tobacco use and have fewer comorbidities [3]. Advances in oncologic therapy have further improved survival in this population, resulting in a growing cohort of patients who now live for decades beyond their cancer diagnosis [15]. This amplifies the long-term impact of treatment-related late effects such as R-ABF and underscores the urgent need for prediction tools and anticipatory management algorithms capable of enabling timely diagnosis and intervention before irreversible autonomic injury accrues. We encourage centers caring for HNC survivors to consider similar multidisciplinary programs, using severity grading to (i) guide individualized therapy, (ii) monitor treatment response using reproducible metrics, and (iii) establish infrastructure for prospective trials aimed at prevention and disease modification, particularly where early detection may permit partial reversibility of baroreflex injury.

Ambulatory 24-hour blood pressure monitoring may reveal characteristic patterns of R-ABF, including alternating hypertensive and hypotensive episodes during the day and absence of nocturnal dipping, often reflecting clinostatic (supine) hypertension, with relatively stable blood pressure at night compared to the labile daytime pattern [14]. Targeted timing of antihypertensive therapy can help mitigate clinostatic hypertension and limit hypertensive surges. Beta-blockers may be added for cardioprotection; however, their use requires careful consideration, as they can theoretically promote unopposed alpha-adrenergic vasoconstriction and paradoxically worsen hypertension [14, 16].

Even when diagnosis occurs late, meaningful symptom improvement remains achievable. Close monitoring and physiology‑guided regimens - including centrally acting α2‑agonists to blunt sympathetically mediated hypertensive surges, paired with agents stabilizing hypotension (such as midodrine or fludrocortisone) - can reduce day‑to‑day variability and improve quality of life when thoughtfully selected and titrated [16]. Our experience challenges the assumption that R‑ABF is invariably refractory due to irreversible carotid sinus or nerve fibrosis. Instead, it suggests that careful agent selection, timing, and iterative titration - guided by autonomic physiology and individualized risk profiles - can materially improve outcomes, even in chronic presentations.

This report reflects a single‑center experience and is inherently limited by its index‑case design and early programmatic data. The diagnostic and therapeutic framework we describe, while grounded in established autonomic physiology, may not be fully generalizable to centers lacking comprehensive autonomic testing or multidisciplinary cardio‑oncology infrastructure. Treatment responses may vary across patients with differing degrees of baroreflex injury, radiation exposure, cardiovascular comorbidities, and medication tolerability. Additionally, the limited duration of follow‑up precludes conclusions about long‑term durability of symptom improvement or whether earlier detection can alter disease trajectory. As a recently established program receiving referrals exclusively from cardiology and oncology clinics, formal pre-test screening data (e.g. screened-to-tested ratios) and systematic therapeutic outcome metrics are not yet available and will be reported as the program matures. These limitations underscore the need for multicenter, prospective studies to refine diagnostic thresholds, validate severity‑grading systems, and define optimal management strategies for R‑ABF.

## Conclusions

R-ABF remains an underrecognized but highly impactful late effect among HNC survivors. This case and early experience from the BARO‑CARE Program demonstrate that systematic autonomic evaluation enables objective diagnosis, severity grading, and physiology‑guided therapy that can meaningfully reduce blood pressure lability and improve quality of life, even when disease is longstanding. Programmatic, multidisciplinary pathways offer a scalable approach to reduce diagnostic delays and standardize care as the survivor population grows. Continued collaborative efforts will be essential to define preventive strategies, optimize treatment algorithms, and improve long‑term outcomes for patients with R‑ABF.

## Supplementary Information

Supplementary Material 1.

## Data Availability

The datasets used and/or analyzed during the current study are available from the corresponding author on reasonable request.

## Abbreviations

ABF: Afferent baroreflex failure
RT: Radiation therapy
HNC: Head and neck cancer
BARO-CARE: Baroreflex Assessment & Rehabilitation for Oncology‑Related Effects Program
SCC: Squamous cell carcinoma
VMAT: Volumetric modulated arc therapy
CASS: Composite autonomic severity score
MSNA: Muscle sympathetic nerve activity
