# Supplementary material for: Targeted autonomic testing for radiation‑induced baroreflex failure in head and neck cancer survivors: index case and early program experience
Source: Cardiooncology. 2026 May 29;12:68. doi: 10.1186/s40959-026-00501-2 (PMC13220401; doi:10.1186/s40959-026-00501-2)
Supplement: Supplementary file 1 — Supplementary Material 1. [file 40959_2026_501_MOESM1_ESM.docx]

**Autonomic Testing Protocol**

**1. Screening of Patients (at least 24 hours prior to scheduled testing):**

- No caffeine, nicotine, alcohol for 12 hours prior to testing.
- Make a note of medications that interfere with autonomic nervous system: beta blockers
- No meal for the last 8 hours.
- Adequate hydration and good rest the day before the test.

**2. Place IV line for labs (norepinephrine)**

**3. Resting phase (at least 10 minutes)**

- Connect patient to device
- Lay on exam table in a quiet room
- Avoid excessive talking

**4. Obtain baseline vital signs on Nova device**

**5. Draw blood for baseline norepinephrine level**

**6. Perform Valsalva Maneuver**

- Keep patient in supine position.

- Practice maneuver once or twice prior to recording

- Instruct the subject to take a deep breath.

- The subject should then forcefully exhale into an Ambu disposable pressure manometer.

- Ensure there is good seal around the mouthpiece with no air leak.

- Maintain expiratory pressure of 40 mmHg (or higher) for 15 seconds. Note if that cannot be achieved.

- Give the subject feedback on the pressure level and time remaining to ensure compliance.

- Minimize Movement and Muscle Activity: Encourage the subject to relax their muscles as this can affect the test results.

- Perform the Valsalva maneuver 3 times (minimum).

- NOTE: For subjects who have a "flat top" response (a reduced or absent early phase II blood pressure response), repeat the maneuver while tilted to 20° or 40° until a significant fall in blood pressure is achieved.

Interpretation:

Key parameters for evaluation include:

- - Valsalva ratio: The ratio of the maximal heart rate during or after the maneuver to the minimal heart rate during the maneuver.
  - Blood pressure changes: Assess the maximal drop in mean blood pressure during phase II, the peak blood pressure at the end of late phase II, and the overshoot in phase IV.
  - Pressure recovery time: The time it takes for systolic blood pressure to return to baseline after phase III.

**Deep Breathing Maneuver:**

Ensure medications (especially those with anticholinergic activity) are withheld for a sufficient time before testing. Caffeine, nicotine, and alcohol should also be avoided prior to testing.

Instructions: Instruct the patient to inhale for 5 seconds and exhale for 5 seconds.

This creates a 10-second respiratory cycle, repeated 6 times. Total duration 1 minute.

Ensure the breathing is continuous and regular, like a wave.

To avoid hyperventilation, breathing should preferably be done through the nostrils with a closed mouth.

Interpretation:

1. Calculate Respiratory Sinus Arrhythmia (RSA): Calculate RSA, which is the difference in heart rate between the end of expiration and the end of inspiration.
2. Average RSA Amplitudes: Average the RSA amplitudes across the 6 respiratory cycles.

Note: The deep breathing test primarily assesses cardiovagal (parasympathetic) function, which is mediated by the vagus nerve. However, the measured heart rate variability (HRV) is influenced by both parasympathetic and sympathetic activity.

**Active Standing:**

Quiet and Temperature-Controlled Environment: The testing room should be quiet and comfortable, with a stable temperature (e.g., 21-23°C). Extreme temperatures can influence blood pressure and heart rate responses.

Supine Rest: The patient should lie down comfortably for at least 5-10 minutes prior to starting the test to establish a baseline heart rate and blood pressure.

The Stand: After the baseline period, the patient should be instructed to stand up quickly but steadily. They should remain as still as possible while standing, avoiding unnecessary movements like tensing muscles or talking, to minimize interference with the results.

Duration and Measurements: The active standing test typically involves remaining in the upright position for 3 to 10 minutes.

Symptom Assessment: The patient should be asked to report any symptoms they experience, such as dizziness, lightheadedness, or nausea, during the test.

Prepared for Symptoms: Be prepared for the possibility of symptoms like lightheadedness, weakness, or even fainting (pre-syncope). If pre-syncope or syncope occurs, the test should be terminated by returning the patient to a lying down position.

Post-Test Monitoring: After the standing portion is completed, the patient should remain supine for a period of time (e.g., 15-20 minutes) while heart rate and blood pressure continue to be monitored.

Interpretation:

- Orthostatic Heart Rate Increase: A significant increase in heart rate (e.g., 30 bpm or more in adults) during the first 10 minutes of standing may be indicative of POTS.
- Blood Pressure Changes: Fluctuations in blood pressure, including a significant drop in systolic or diastolic blood pressure, are assessed. A decline in systolic BP greater than 20 mmHg and diastolic BP greater than 10 mmHg after standing is considered abnormal.
- Symptom Correlation: Symptoms experienced during the test are correlated with the observed heart rate and blood pressure changes.

**Cold Pressor Maneuver**

Ensure the subject is rested and comfortable. They should be seated and relaxed for a period of time before the test, typically 15-20 minutes. Avoid stimulants like caffeine and nicotine for at least 3-4 hours prior, and alcohol for 8 hours. Sympathomimetic and anticholinergic medications should ideally be stopped for 24-48 hours before testing, if clinically permissible.

Use a container (e.g., bucket, tub) large enough to fully immerse the hand or forearm. It should be filled with ice water. A temperature of 4°C is often used.

Baseline Measurements: Record baseline BP and HR measurements for several minutes (e.g., 30 minutes) before the cold pressor test.

Instruct the subject to immerse their hand or forearm in the ice water up to a consistent depth (e.g., 5 cm above the wrist).

Ensure the hand is relaxed and the palm is facing up.

Ensure no metal parts of the container touch the hand, as this can affect heat transfer.

Duration: The immersion duration is typically 1-3 minutes. A common duration is 60-90 seconds.

Primary Outcome Measures: The main outcomes are the change in systolic and diastolic BP and HR in response to the cold stimulus.

Screen for Contraindications: Assess subjects for conditions that might make the test unsafe, such as a history of cardiovascular disease, fainting, seizures, or Raynaud's phenomenon.

Monitor for Adverse Reactions: Watch for signs of syncope (fainting) or other adverse reactions.

Activation of afferent sensory pathways by the cold stimulus triggers a sympathetic response leading to increased BP and HR.

- **Positive Result:**

A significant increase in blood pressure (≥15 mmHg increase in SBP, DBP, or MAP) indicates a positive response, suggesting a normal sympathetic response to the cold stimulus.

- **Negative Result:**

A smaller increase in blood pressure (<15 mmHg) indicates a negative response, which may suggest issues with the central nervous system, or efferent sympathetic pathways.

A normal response in the CPT, despite other abnormal responses (like in a Valsalva maneuver), may indicate a lesion in the baroreceptor afferent pathway.

In afferent carotid baroreflex failure, the cold pressor test typically shows an exaggerated response.

**Norepinephrine level testing:**

Medication Withdrawal: Discontinue any medications that can affect catecholamine levels (e.g., antidepressants, decongestants, certain antihypertensives) for at least one week prior, if medically feasible.

Fasting: Ensure the patient fasts overnight, or for a specified duration (at least 4 hours) before the test. Dietary influences, such as caffeine and certain foods containing biogenic amines, can significantly impact results.

Avoid Stimulants: Instruct the patient to avoid caffeine, nicotine, and strenuous exercise before the test.

Rest and Relaxation: Have the patient rest in a quiet, supine position for at least 30 minutes before blood collection. Stress, including difficult venipuncture, can increase catecholamine levels.

Indwelling Catheter: Use an indwelling intravenous catheter to minimize venipuncture-related stress and ensure consistent blood collection without repeated needle sticks.

Supine Position: Draw blood while the patient remains in the supine position after the resting period. Upright posture increases sympathetic activation and can lead to elevated levels.

Appropriate Collection Tube: Collect the blood in a chilled tube containing EDTA-sodium metabisulfite solution. This helps stabilize catecholamines.

Immediate Chilling and Processing: Place the tube on ice immediately after collection and process it rapidly (within 30 minutes) in a refrigerated centrifuge to separate the plasma.

Baseline (Supine) Levels:

Normally, serum norepinephrine levels are measured in a supine position before any postural change.

Low baseline norepinephrine can suggest postganglionic sympathetic denervation.

In Pure Autonomic Failure (PAF), baseline norepinephrine levels are often very low.

Orthostatic (Upright/Tilt) Response:

A healthy response to standing or head-up tilt includes an increase in plasma norepinephrine levels, reflecting sympathetic activation.

Failure of norepinephrine to rise during upright posture (such as during a head-up tilt test) is considered a diagnostic sign of autonomic failure, especially in patients with postural hypotension.

Attenuated or absent orthostatic increase in norepinephrine can indicate pre- or postganglionic sympathetic lesions.

In Postural Orthostatic Tachycardia Syndrome (POTS), patients may have high plasma norepinephrine levels during orthostasis, which can be associated with increased heart rate and blood pressure.

Other Considerations:

Elevated norepinephrine levels may be associated with hypertension, stress, anxiety, or certain medications.

Extremely high norepinephrine levels may suggest a pheochromocytoma, a rare adrenal tumor that can cause severe hypertension.

In specific forms of autonomic neuropathy like pandysautonomia, basal norepinephrine levels are low and don't rise with head-up tilt testing.

However, it's important to note that changes in norepinephrine levels during postural changes may not always be solely due to sympathetic activation; decreased norepinephrine clearance can also play a role.
